# Percutaneous transhepatic stent for chronic intestinal bleeding from jejunal varices in primary idiophatic superior mesenteric vein stenosis: A case report^☆^

**DOI:** 10.1016/j.radcr.2022.01.031

**Published:** 2022-02-12

**Authors:** Renato Argirò, Leonardo Vattermoli, Francesca Di Pietro, Sara Crociati, Luca Funari, Valentina Perlangeli, Roberto Floris

**Affiliations:** a Interventional Radiology Unit, Department of Biomedicine and Prevention, University of Rome "Tor Vergata", Viale Oxford 81, Rome, 00133, Italy; b Diagnostic Imaging Unit, Department of Biomedicine and Prevention, University of Rome Tor Vergata, Viale Oxford 81, Rome, 00133, Italy; c Neuroradiology Unit, Department of Biomedicine and Prevention, University of Rome "Tor Vergata", Viale Oxford 81, Rome, 00133, Italy; d Neurology Unit, Department of Neurology, University of Rome Tor Vergata, Viale Oxford 81, Rome, 00133, Italy

**Keywords:** Interventional radiology, Varices, Transhepatic stent, GI bleeding, Vein stenosis

## Abstract

Jejunal varices are a rare cause of gastrointestinal bleeding. In most cases, they are due to portal hypertension related to liver cirrhosis, less frequently to superior mesenteric vein stenosis (SMV). In this article we describe an unusual case of a 61 year-old male patient who arrived at our emergency department with intermittent variceal bleeding due to jejunal varices causing melena and subsequent chronic anaemia. Patient was indeed discovered to have primary idiopathic superior mesenteric vein stenosis. We managed to treat this patient via SMV stenting through percutaneous transhepatic approach. In cases of upper-GI bleed with negative endoscopy for active bleeding, a contrast-enhanced CT scan should be performed to diagnose jejunal varices and their underlying cause, such as SMV stenosis which is best treated with percutaneous phlebography.

## Introduction

Jejunal varices are an infrequent cause of gastrointestinal bleeding and represent a challenging diagnosis for their uncommon location mostly because of their low prevalence. Such varices are secondary to portal hypertension in cirrhosis or are less frequently caused by extrahepatic portal vein occlusion or thrombosis [1], [2], [3], [4], [5], [6], [7].

Similar to extra-hepatic portal vein occlusion, last but noy least the origin of small bowel varices could be a SMV stenosis which leads to thrombosis, either acute or chronic [8], [9], [10], [11].

These two similar entities share multiple aetiologies and can be the result of post-operative adherences after duodenocephalopancreasectomy or liver transplant [3,12], external compression from pancreatic head tumours [12,13] mid-gut carcinoid [14], pancreatitis, Crohn's disease [15] and even in patients with rare inherited coagulation disorders [2].

Symptomatic jejunal varices can be treated with endoscopy or surgery, but it is always basic to treat the underlying splanchnic hypertension [1,^5^,^8^,11].

In case of SMV stenosis, possible treatments include surgery or percutaneous transcatheter recanalization with angioplasty and stent placement [8,^9^,[12], [13], [14],16].

We describe an unusual case of idiopathic SMV stenosis in a patient with no history of pancreatic or hepatic diseases or major abdominal surgery, who had development of jejunal varices with chronic gastrointestinal bleeding which led to melena and anaemia. We treated patient with percutaneous transhepatic SMV stent placement.

## Case report

A 61 years-old male patient was admitted to our hospital with melena, abdominal pain and blood loss-related symptoms like paleness and faintness. His blood tests showed his haemoglobin (Hb) was as low as 4.9 g/dL, RBC 2.25 million/µL, HT at 16.5%, high RDW and low MCH and MVC values.

Patient was transfused with multiple units packed RBCs and underwent EGDS examination, which revealed distal duodenum mucosa hyperaemia with violaceus spots but no active bleeding, leading to suspect intestinal ischaemia.

**Fig. 1:**
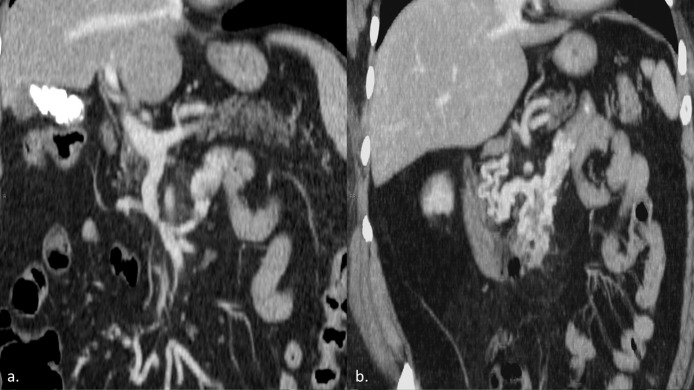
Diagnostic CT-scan. Contrast enhanced CT-scan showed pre-occlusive and focal stenosis at the distal portion of the SMV (a), with swollen varicoid venous plexes around the pancreatic head, the duodenum and proximal jejunum (b),

The following contrast enhanced CT-scan showed an abnormally expanded SMV with pre-occlusive and focal stenosis at its distal portion, bypassed by multiple varicoid venous plexuses around cephalic pancreas, duodenum and proximal jejunum (Fig 1 a-b) The portal vein was enlarged (17 mm) with no CT signs of cirrhosis, ascites or pancreatic abnormalities. Indeed patient had no history nor CT evidence of previous major abdominal surgery, pancreatitis, chronic hepatopathy or cancer.

After an urgent multidisciplinary meeting, our team chose a percutaneous trans-hepatic approach to reduce pressure in the varices.

In the interventional suite after local anaesthesia and under US-guidance, we identified a sub-segmentary portal branch in segment 5 (V) and we performed puncture with a micro-puncture set (21 Gauge needle and 0.018′’ guide-wire). SMV was catheterised after insertion of a 6 Fr vascular sheath into the portal vein.

Navigation across the stenosis was achieved with a 4 Fr Bern catheter (Cordis) coupled with 0.018′’ hydrophilic guide-wire (Terumo Advantage).

**Fig. 2:**
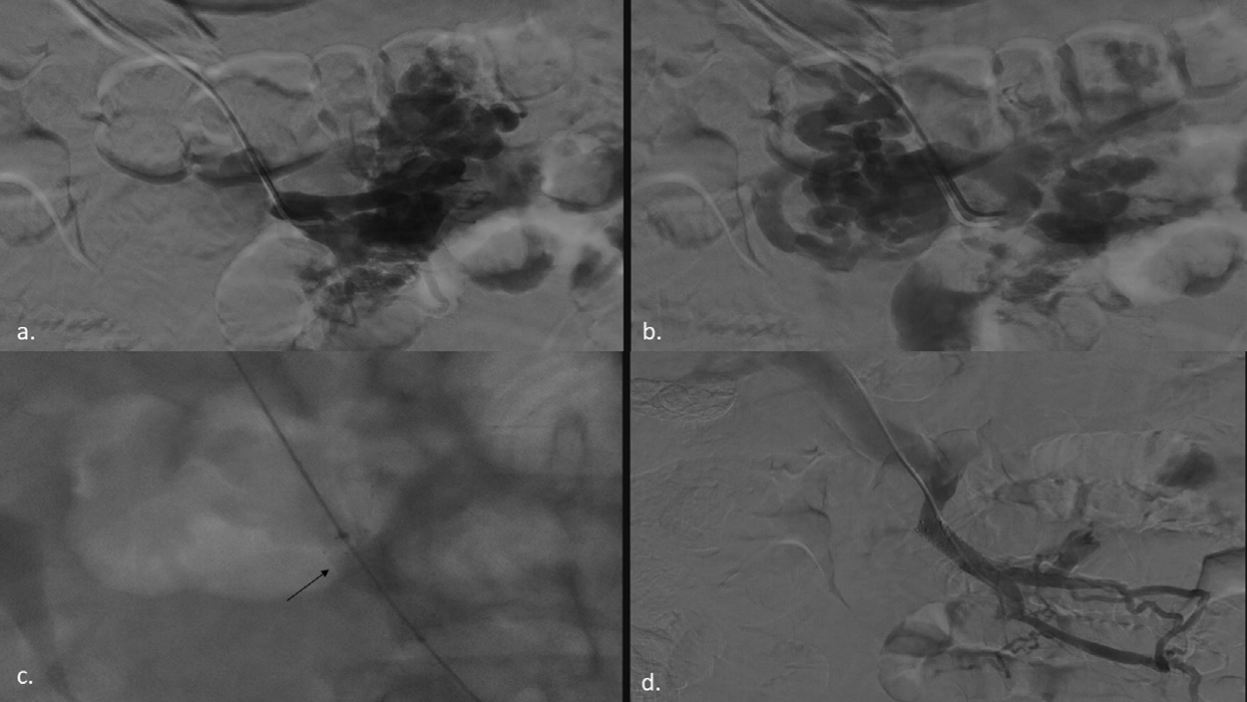
Percutaneous phlebography and stent placement. Percutaneous transportal phlebography showed opacification of the duodenal and jejunal varices (a-b) with late opacification of the pre-stenotic portion of SMV (A). Balloon-expandable stent (arrow) placement to cover the entire stenotic segment (c). Phlebography performed subsequently showed resolution of the stenosis and duodenal, pancreatic and jejunal varices decompressed (d),

Phlebography was performed confirming duodenal and jejunal varices opacification. Late filling of pre-stenotic portion of SMV was observed (Fig 2 a-b).

Invasive intravenous pressure was measured in the pre-stenotic and post-stenotic tract showing high pressure gradient, 34 mmHg in the first one vs 27 mmHg in the portal vein.

After switching the guide-wire from 0.018′’ to 0.035′’, a 10 × 27 mm balloon-expandable stent was placed to cover the entire stenotic segment (Fig 2 c).

Phlebography was performed again to demonstrate resolution of the stenosis and decompression of duodenal, pancreatic and jejunal varices (Fig 2 d).

Invasive intravenous pressure measurement showed resolution of the pressure gradient with a pressure of 28 mmHg in the mesenteric vein and 25 mmHg in the portal one.

The access portion of liver parenchyma was embolized during sheet retrieve with Lipiodol and Glubran with 1:1 dilution, without post-procedural bleeding.

On first post-operative day (POD), anti-platelet therapy was set as daily 100 mg Acetylsalicylic acid.

Blood tests before discharge (on third POD) showed an improved Hb (9.9 g/dL), RBC count (3.77 million/µL) and Ht (31.6%).

**Fig. 3:**
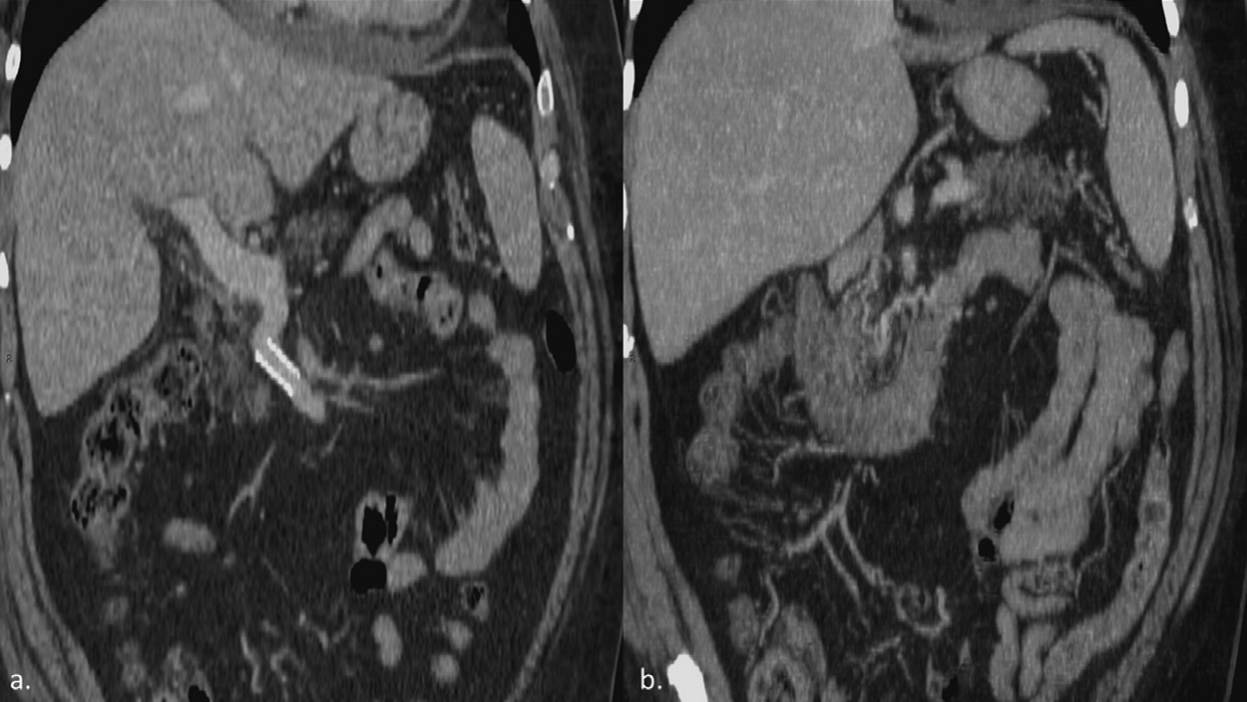
One month control CT-scan. Contrast-enhanced CT scan performed after one month showed SMV stent patency (a) and resolution of jejunal collateral varicosities (b).

Control CT-scan performed one month later confirmed procedure results, showing patency of SMV stent and mainly there was no more evidence of duodenal and jejunal collateral varicosities (Fig 3 a-b).

At long-term follow-up (3, 6 and 12 months) patient maintained optimal blood tests range with no sign of anemization or clinical evidence of melena.

## Discussion

Our clinical case represents a typical chronic gastrointestinal blood loss, secondary to a rare pathological entity like jejunal varices. Generally they are consequence of splanchnic venous hypertension [1], [2], [3], [4], [5], [6].

Indeed, jejunal varices share other common gastrointestinal varices pathogenesis, with chronic obstruction of normal hepatopetal blood outflow through the portal system, causing a retrograde venous flow into collateral vascular systems in peripancreatic e periduodenal regions, as seen in our case, with increased venous pressure as well as size.

Chronic splanchnic venous hypertension is basically due to intrahepatic portal vein obstruction (e.g. cirrhosis) or to extrahepatic splanchnic veins stenosis or chronic thrombosis. These conditions typically involve the portal vein but in some rare cases, like this one, also the SMV.

Even though cases of anomalous development of the inferior vena cava are reported in literature [18], SMV stenosis is not frequent and there is evidence of few documented cases. Frequent etiologies are: acute thrombosis evolving into chronic thrombosis [17]; complicated pancreatitis or other duodenal and biliary inflammatory processes; surgical procedures involving pancreas, duodenum or hepatobiliary system (e.g. Whipple procedure, Rou-en-Y bypass or hepatic transplant); malignant infiltration from pancreatic cancers or mid-gut tumours like carcinoids [8,[12], [13], [14]. Singular cases of SMV stenosis or thrombosis caused by hereditary coagulation disorders [2] and Crohn's disease [15] have also been reported.

Our case showed not only the presence of two rare clinical entities, jejunal varices and SMV stenosis, but also the idiopathic nature of this stenosis, since the patient didn't have any history of pancreatic or biliary inflammatory disease, nor pancreatic, hepatobiliary or gastrointestinal surgical procedures, or abdominal oncological diseases. Furthermore no possible explaining abnormalities were found after CT scan and EGDS.

Patients developing jejunal varices are usually asymptomatic. A rare but typical clinical manifestation consists in gastrointestinal bleeding, which could be more or less severe in unusual acute hematemesis. This is a life-threatening situation more frequently observed in gastroesophageal varices, or typically in form of melena, with related chronic blood loss symptoms like anaemia and weakness, as we observed [1,^3^,[5], [6], [7], [8].

Obviously, symptoms are often related to the underlying pathology that causes jejunal varices. In a case of clinically significative SMV stenosis, that is normally asymptomatic, not specific signs and symptoms of splanchnic venous hypertension can be observed: recurrent ascites, splenomegaly, chronic unlocalizable abdominal pain, bowel congestion that can end with bowel ischemia [12,17].

In our case, diagnosis of jejunal varices was not achieved by endoscopic examination, even though it is the first exam performed in case of melena or gastrointestinal bleeding, due to their distal location. The gold standard for jejunal varices diagnosis is contrast-enhanced CT-scan, since it detects precisely collaterals vessels, active bleeding and underlying vascular triggering aetiology like splanchnic venous occlusion or stenosis, including SMV stenosis and hypertrophic collateral porto-systemic circulation [1], [2], [3], [4], [5], [6], [7], [8].

CT-scan is also useful to identify the aforementioned plausible causes of SMV stenosis and to evaluate some possible congestion or small bowel ischemia [12,17].

In a splanchnic vein obstruction seen for the first time in CT-scan, next diagnostical step is trans-portal phlebography. This procedure can exactly define both the localization and the grade of stenosis. So operator is able to: a. measure venous pressure gradients before and after the stenotic tract; b. study blood flow direction of whole splanchnic venous system, discovering possible retrograde flows into portosystemic collaterals, as observed in the case reported, allowing direct endovascular recanalization [1,^4^,^5^,[7], [8], [9],[11], [12], [13], [14], [15], [16].

Jejunal varices can be treated surgically, through excision of the duodenal or jejunum tract involved or with creation of a vascular shunt to bypass the splanchnic venous stenotic tract [1,^3^,^8^,10], even though the most used treatment consists in an endovascular approach to obtain recanalization of the occluded/stenotic vein as a means to resolve venous hypertension.

Similar to portal vein thrombosis with jejunal varices development, many authors reported some cases of SMV stenosis treatment brought about malignant oncological or post-surgical cause. They observed complete regression of varices and portal hypertension, less complications and gastrointestinal rebleeding as compared to surgical approaches [7], [8], [9],[11], [12], [13], [14],16].

Percutaneous procedure can cause bleeding from transhepatic access point. This complication could be avoided with biocompatible glue injection in the percutaneous path.

Indeed, we successfully treated SMV stenosis through percutaneous trans-portal stent positioning, restoring vascular lumen diameter, venous pressure and flow into collateral circulation. It means significant reduction of the jejunal varices, without any relevant complications or other gastrointestinal bleeding episodes over the following 12 months.

## Conclusion

Jejunal varices are a rare but possible cause of gastrointestinal bleeding. Differential diagnosis should always be considered especially in patients with risk factors for portal or mesenteric hypertension. Even if they are mainly secondary to portal vein thrombosis or stenosis, a possible uncommon cause can be represented by isolated superior mesenteric vein stenosis. Diagnosis of jejunal varices requires contrast-enhanced CT-scan. Another useful diagnostic tool for SMV stenosis is evaluation with portal-mesenteric angiography. Primary and more indicated treatment of jejunal varices secondary to mesenteric vein stenosis is endovascular recanalization through stent placement.

## Acknowledgments

None.

## Conflict of interest

The authors declare that they have no financial activities related to the present article.

The authors declare that they have no known competing financial interests or personal relationships that could have appeared to influence the work reported in this paper.

## Patient Consent

To whom it may concern, hereby we confirmed that we have obtained the written informed patient consent for the publication of this case report.

Rome, 01/11/2021

Dr. L. Vattermoli and the other authors
